# 

**Published:** 1889-01-12

**Authors:** 


### CONTENTS.

**Eyes and Sight** - - - - - - - - 227

**Within the Wards.**—A New Suggestion about Nerve Centres 228

**The Doctor's Pulpit.**—"All Sorts and Conditions" of Women - - - - - - - - 229

**Measles** - - - - - - - - 230

**Glances at the Insane in Other Lands.** By a Medical Superintendent.—III. Pennsylvania - - - 231

**Voyages in Vacation.**—XIV. The Kremlin, Moscow - 233

**Notes and News** - - - - - - - 234

**Everybody's Page** - - - - - - - 236

**Annotations.**—The *Lancet* and the National Pension Fund—Vicarious Digestion—Coughing in Church: a Defence - - 237

**Babies.**—II. More about the World's Babies - - 238

**Metropolitan Hospitals Ancient and Modern.**—III. The French System—Medical Schools - - - 239

**False Impressions.** By Caroline Fothergill, Author of "An Enthusiast," "A Voice in the Wilderness," etc. - 240

**"Failed"** - - - - - - - - 241

**Scraps and Gleanings** - - - - - - - 242

**Amusements and Relaxation** - - - - - 242

### EXTRA SUPPLEMENT—THE NURSING MIRROR.

**En Passant.**—Short Items—Kindly Greeting—Religious or Lay Nurses—Glasgow Royal Infirmary—To the Mission Field—Probationers a Blessing—Novel Nurses - - - lvii
**Original Article.**—Lady Guardians - - - - lviii
**National Pension Fund for Nurses** - - - - lix
**The Bradford Infirmary** - - - - - - lix
**A Christmas Holiday.**—Part I. - - - - - lix
**Everybody's Opinion.**—Hospital Sweating—Medical and Surgical Homes: Fifteen Years' Experience—Rest and Exercise lx
**Vacancies.—Notes and Queries** - - - - - lx

